# Dedifferentiated liposarcoma presenting with ascites as the initial clinical manifestation: a case report and literature review

**DOI:** 10.3389/fmed.2026.1862667

**Published:** 2026-05-29

**Authors:** JingJun Wang, LiQian Chen, MaoTao He, MaiQing Yang

**Affiliations:** 1 Department of Obstetrics, Changyi People’s Hospital, Changyi, Shandong, China; 2 Department of Pathology, Weifang People’s Hospital (First Affiliated Hospital of Shandong Second Medical University), Weifang, Shandong, China

**Keywords:** amplification, ascites, dedifferentiated liposarcoma, diagnosis, fluorescence *in situ* hybridization, immunohistochemical staining, mouse double minute 2 homolog

## Abstract

Dedifferentiated liposarcoma (DDLPS) is a high-grade soft tissue sarcoma predominantly arising in the retroperitoneum, characterized by an insidious clinical course. This study reports a 63-year-old female patient who presented with abdominal distension and decreased appetite, without other systemic complaints. Malignant tumor cells were identified in both ascitic smears and cell blocks. Combined with immunohistochemical (IHC) staining, the findings were consistent with sarcoma cells. Fluorescence *in situ* hybridization (FISH) analysis of the tissue biopsy revealed clustered amplification of mouse double minute 2 homolog (MDM2) gene. Based on the combined results of IHC and FISH testing, the final pathological diagnosis of the tissue biopsy was DDLPS. To the best of existing knowledge, this is one of the few reported cases of DDLPS initially manifesting with malignant cells detected in peritoneal fluid. This case highlights the diagnostic challenge of rare sarcomas presenting with serosal effusions and emphasizes the importance of integrating cytopathological, radiological, and molecular testing for accurate diagnosis and optimal management. Preventing misdiagnosis of this tumor as other histological subtypes is of great clinical significance, while IHC features and FISH can provide important clues for its accurate diagnosis.

## 1 Introduction

Dedifferentiated liposarcoma (DDLPS) accounts for 10–15% of all liposarcomas and represents a high-grade transformation of well-differentiated liposarcoma, most commonly occurring in the retroperitoneum, followed by the extremities and mediastinum (1, 2). DDLPS exhibits aggressive biological behavior, with high local recurrence rates and potential for distant metastasis, and the 5-year overall survival rate ranges from 30 to 50% (3–5). Classic clinical presentations of DDLPS include abdominal pain, palpable abdominal mass, bowel obstruction, or weight loss; however, initial manifestation as malignant peritoneal cytology is extremely rare (6–9).

The DDLPS diagnosis in ascitic fluid is particularly challenging due to the non-specific cytomorphology of malignant cells, which may mimic carcinoma, malignant mesothelioma, or other sarcoma subtypes (7). Definitive diagnosis relies on immunohistochemical (IHC) markers and molecular confirmation of mouse double minute 2 homolog (MDM2) gene amplification, which is the pathognomonic feature of DDLPS (3, 10). This case underscores the need for a high index of suspicion and comprehensive evaluation—including cytopathology, radiology, and molecular testing—in patients with unexplained malignant ascites, especially when no primary epithelial tumor is identified.

## 2 Case presentation

### 2.1 Clinical history

A 63-year-old female was admitted to the hospital presented with abdominal distension and decreased appetite for 10 days. The patient’s general state was normal. The patient had undergone partial pulmonary resection 5 years earlier for minimally invasive adenocarcinoma of the lung. No family history was reported. Diagnostic paracentesis was performed, yielding 500 mL of serosanguineous fluid. Cytological examination revealed malignant tumor cells. A cell block was prepared for further IHC analysis. Contrast-enhanced computed tomography (CT) showed a patchy soft tissue density anterior to the left iliopsoas muscle, measuring approximately 4.6 cm in diameter, with obscuration of the surrounding fat planes. On contrast-enhanced imaging, the lesion showed heterogeneous ring enhancement with internal patchy non-enhancing low-density areas, and is closely adjacent to the left common iliac artery. A bulky soft tissue mass was noted along the left pelvic sidewall, approximately 7.0 cm in diameter, demonstrating heterogeneous enhancement. Multiple masses were identified anterior to the left iliopsoas muscle and within the left pelvic cavity (Figure 1).

**Figure 1:**
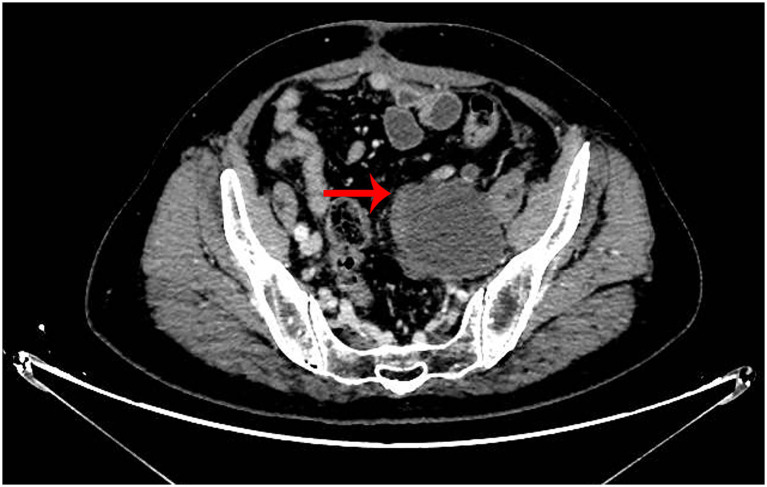
Computed tomography of the pelvis. A patchy soft tissue density was seen anterior to the left iliopsoas muscle, measuring approximately 4.6 cm in diameter, with obscuration of the surrounding fat planes. On contrast-enhanced imaging, the lesion showed heterogeneous ring enhancement with internal patchy non-enhancing low-density areas, and was closely adjacent to the left common iliac artery. A bulky soft tissue mass was noted along the left pelvic sidewall, approximately 7.0 cm in diameter, demonstrating heterogeneous enhancement. Multiple masses were identified anterior to the left iliopsoas muscle and within the left pelvic cavity.

### 2.2 Immunohistochemical staining and fluorescence *in situ* hybridization

Ascites (500 mL) was centrifuged at 6,000 × g for 10 min. The supernatant was discarded; the cell pellet was smeared on slides and processed for hematoxylin and eosin (HE) staining. For cell blocks, the pellet was re-centrifuged at 6,000 × g for 10 min, wrapped in filter paper, dehydrated, paraffin-embedded, and sectioned at 4-μm for immunohistochemistry.

The biopsied specimens were fixed in 10% neutral-buffered formalin, paraffin-embedded, and sectioned at 4-μm. HE staining was used for histological evaluation. Tumor sections were immunostained with available primary antibodies against cytokeratin 7 (CK7), CK-pan, vimentin, P16, smooth muscle actin (SMA), desmin, cyclin-dependent kinase 4 (CDK4), CD34, S-100, CK5/6, Wilms tumor-1 (WT-1), calretinin, and Ki-67 (Maixin, Fuzhou, China). Immunohistochemistry was performed using EnVision. Negative and positive controls were included in the IHC assay.

The fluorescence *in situ* hybridization (FISH) testing for MDM2 gene amplification was performed using a commercial kit (Kanglu Biotechnology Co., Ltd., Wuhan, China). Assays were conducted on 4-μm thick tissue sections, following the manufacturer’s protocol. FISH signals were examined under a fluorescence microscope with appropriate filters, demonstrating bright yellow-green hybridization signals in counterstained nuclei. FISH was performed using an MDM2/CEP12 dual-color probe set. The ratio of MDM2 to CEP12 signals was calculated in each nucleus. A ratio ≥2.0 was defined as MDM2 amplification (positive). Cases with a ratio <2.0 were considered negative. Additionally, clustered MDM2 signals within the nucleus were also regarded as indicative of gene amplification, even when the ratio was less than 2.0.

### 2.3 Pathological diagnosis and follow-up history

The tumor specimen was embedded in a paraffin block and examined. Cytological examination revealed single malignant cells exhibiting marked nuclear pleomorphism, prominent nucleoli, irregular nuclear membranes, abundant cytoplasm, and occasional multinucleation (Figures 2A–D). The cells showed epithelioid morphology, with no glandular or squamous differentiation, ruling out metastatic carcinoma. IHC results of cell block showed positive staining for vimentin, and negative for CK-pan (Figure 3A), CK7, S-100, CK5/6, WT-1, calretinin, CD34, and SMA. Combined with IHC staining, the cytological findings were consistent with sarcoma cells. Due to the limited number of tumor cells in the cell block, further studies could not be performed, so a tissue needle biopsy was performed to clarify the subtype. Pathological examination of biopsy revealed a spindle cell tumor with marked nuclear pleomorphism, moderate atypia, and interstitial collagenous hyperplasia accompanied by myxoid degeneration (Figures 2E,F). IHC results showed positivity for vimentin, P16 (Figures 3B,C), SMA, and CDK4. And negative for CK-pan, Desmin, CD34, and S-100. The Ki-67 proliferation index was approximately 20%. FISH analysis showed clustered amplification of the MDM2 gene (Figure 3D). Combined with IHC and FISH findings, the features were consistent with DDLPS.

**Figure 2:**
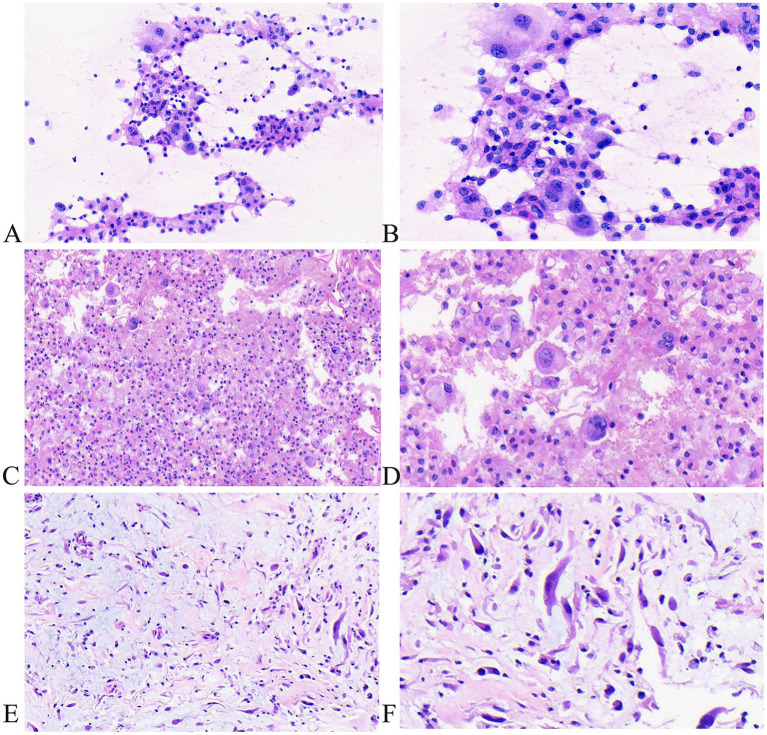
Histological features of the tumor. **(A,B)** Cytology smears: **(A)** Microscopically, scattered, single neoplastic cells with intact morphology, abundant cytoplasm (hematoxylin and eosin, ×100). **(B)** Tumor cells had increased nuclear-to-cytoplasmic ratio, and prominent nucleoli, allowing clear assessment of cellular atypia (hematoxylin and eosin, ×200). **(C,D)** Cell blocks: **(C)** Showed individually scattered cells (hematoxylin and eosin, ×100) **(D)** Revealed neoplastic cells with abundant cytoplasm, distinct nucleoli, and marked nuclear atypia (hematoxylin and eosin, ×200). **(E,F)** Biopsy specimens: **(E)** Showed neoplastic cells infiltrating a myxoid background; high-power view (hematoxylin and eosin, ×100). **(F)** Clearly demonstrated spindle-shaped neoplastic cells with abundant cytoplasm, hyperchromatic and irregular nuclei, and marked atypia (hematoxylin and eosin, ×200).

**Figure 3:**
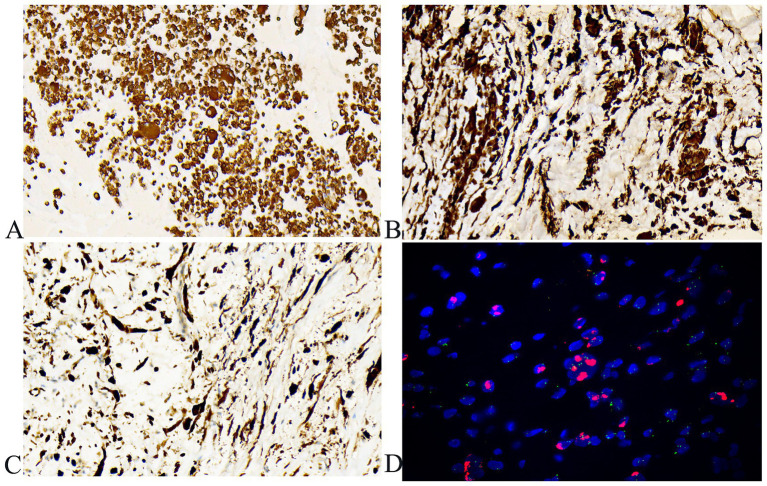
Immunohistochemistry and fluorescence *in situ* hybridization of dedifferentiated liposarcoma. Immunohistochemically, the tumor cells of cell blocks showed positive expression of vimentin **(A)**. Tumor cells of biopsy specimens showed positive expression of vimentin **(B)** and P16 **(C)** (×100). Fluorescence in situ hybridization showed clustered amplification of the MDM2 gene **(D)** (×400).

The patient was examined by a multidisciplinary sarcoma team comprising medical oncologists, surgical oncologists, and pathologists. Given the presence of peritoneal seeding and the patient’s preference, radical surgical resection was considered not feasible. The patient was treated with chemotherapy consisting of docetaxel plus gemcitabine, followed by maintenance therapy with oral anlotinib. However, the patient died after 26 months of follow-up.

## 3 Discussion

DDLPS is a rare, aggressive soft tissue sarcoma with a predilection for the retroperitoneum, where its deep location often delays diagnosis until the tumor is large enough to cause mass-effect symptoms (11–13). Isolated massive ascites as the initial presenting sign is extremely uncommon, as sarcomatous peritoneal involvement typically occurs late in the disease course, after local invasion or distant metastasis. Only a few case reports describe DDLPS presenting with ascites, and even rarer are those that identify malignant cells in peritoneal fluid as the initial diagnostic clue (7, 9, 14).

Six cases of DDLPS diagnosed by ascitic fluid cytology were reviewed, including five published cases (2021–2024) and one ongoing case (2026). Patients included three men and three women, aged 47–70 years (median 61 years). Tumor cells were predominantly single with occasional clusters, showing marked pleomorphism, epithelioid features, coarse chromatin, prominent nucleoli, and multinucleation. Treatment included chemotherapy (4 cases), surgical resection (1 case), and radical resection plus adjuvant radiotherapy (1 case). Two patients were disease-free, one was alive with disease but lost to follow-up, and three died of disease at 25–26 months, indicating a generally poor prognosis (7, 9, 14) (Table 1).

**Table 1:** Summary of dedifferentiated liposarcoma cells in ascitic fluid.

No.	Year	Sex	Age	Cellularity	Cellular arrangement	Cytological features	Therapy	Outcome
1 (9)	2021	M	61	NA	Single cells	Marked pleomorphism	Chemotherapy	Alive with disease, lost to follow-up at 21 months.
2 (9)	2021	F	54	NA	Single cells	Marked pleomorphism	Chemotherapy	Dead of disease at 26 months.
3 (14)	2024	F	50	Highly cellular	Single cells, clusters	Atypical adipocytes, multivacuolated lipoblasts	Radical resection with adjuvant radiotherapy	Disease free for 12 years
4 (14)	2024	M	47	Highly cellular	Single cells, clusters	Isolated multivacuolated lipoblasts, nuclear irregularity, nuclear enlargement,	Chemotherapy	Died with disease 25 months
5 (7)	2024	M	70	Moderately cellular	Single cells, clusters	Epithelioid morphology, coarse chromatin, prominent nucleoli, multinucleation, marked pleomorphism	Surgical resection	Disease free for 6 months
6 (present case)	2026	F	63	Poorly cellular	Single cells	Epithelioid morphology, coarse chromatin, prominent nucleoli, multinucleation	Chemotherapy	Died with disease 26 months

NA: not available.

The cytological diagnosis of DDLPS in ascitic fluid is indeed fraught with difficulty, as malignant cells in ascites often exhibit non-specific pleomorphic epithelioid or spindle features, which can easily mimic metastatic adenocarcinoma, malignant mesothelioma, or undifferentiated pleomorphic sarcoma (7, 9, 14). To clarify the differential diagnosis, this study has supplemented a table summarizing the key differences in cellular morphology and immunohistochemical features among these types of tumors (Supplementary Table 1). Key distinguishing features include the absence of glandular differentiation (ruling out carcinoma) and negative mesothelial markers (such as calretinin and WT-1), which exclude malignant mesothelioma. Definitive diagnosis requires demonstration of MDM2 and CDK4 overexpression by IHC, together with MDM2 amplification by FISH, findings that remain pathognomonic for DDLPS and distinguish it from other high-grade sarcomas (5, 10, 15, 16).

This case illustrates three key clinical lessons regarding DDLPS presenting as malignant ascites. First, unexplained malignant ascites with negative epithelial markers should prompt suspicion for rare mesenchymal tumors such as DDLPS. Sarcomas account for less than 6% of malignant effusions, and DDLPS cells frequently mimic carcinoma or mesothelioma, while epithelial and mesothelial markers (such as CK, calretinin, and WT-1) are negative, leading to underdiagnosis (8, 9, 17). Second, ascitic fluid cytology with cell block and targeted IHC is essential for identifying the sarcomatous origin (18). FISH confirmation of MDM2/CDK4 amplification is pathognomonic (10). Third, multidisciplinary correlation including imaging, histopathology, and molecular testing is mandatory for accurate diagnosis. Intratumoral heterogeneity may limit biopsy sampling, whereas effusion cytology can reveal high-grade components (19, 20). Given the poor prognosis of DDLPS with serosal involvement, prompt and precise diagnosis ensures appropriate sarcoma-directed management and avoids ineffective therapies for carcinoma or mesothelioma (8, 16). For advanced unresectable DDLPS, docetaxel plus gemcitabine followed by anlotinib represents an effective and well-tolerated treatment option. In addition to conventional chemo-antiangiogenic therapy, MDM2-targeted therapy and immune checkpoint immunotherapy have emerged as promising alternative strategies. MDM2 inhibitors restore p53 activity and yield durable disease control in MDM2-amplified liposarcoma (21, 22), while immunotherapy, either alone or combined with anti-angiogenic agents, offers potential clinical benefits for refractory cases (23, 24). Further clinical studies are needed to optimize their clinical application in this disease. This case contributes to the limited literature on rare presentations of DDLPS and underscores the importance of a comprehensive diagnostic approach in patients with unexplained malignant ascites.

## 4 Conclusion

In summary, this study reports a rare case of DDLPS initially presenting with massive ascites as the sole clinical manifestation, with malignant cells identified in peritoneal fluid. This case underscores the diagnostic challenges of DDLPS in serosal effusions and highlights the importance of integrating cytopathological, immunohistochemical, and molecular testing for accurate diagnosis. Early recognition of this rare presentation may facilitate timely referral to sarcoma specialists and optimize palliative or therapeutic interventions for affected patients.

## Data Availability

The original contributions presented in the study are included in the article/Supplementary material, further inquiries can be directed to the corresponding author.
