# Supplementary material for: Dedifferentiated liposarcoma presenting with ascites as the initial clinical manifestation: a case report and literature review
Source: Front Med (Lausanne). 2026 May 29;13:1862667. doi: 10.3389/fmed.2026.1862667 (PMC13259791; doi:10.3389/fmed.2026.1862667)
Supplement: Supplementary file 1 [file Table_1.DOCX]

| Tumor type | Cellular Morphology | Immunohistochemical Features |
| --- | --- | --- |
| Dedifferentiated Liposarcoma | Pleomorphic epithelioid or spindle cells; scattered atypical lipoblasts (occasionally visible); nuclear pleomorphism is obvious; cytoplasm is moderate to abundant, sometimes with lipid vacuoles; no glandular or papillary structures. | Vimentin (+), S-100 (+), MDM2 (+), CDK4 (+) in most cases; AE1/AE3 (-), Calretinin (-), WT-1 (-). |
| Metastatic Adenocarcinoma | Epithelioid cells arranged in nests, glands, or acini; nuclear pleomorphism is mild to moderate; cytoplasm is abundant, often eosinophilic or clear; mucus secretion can be seen in some cases; cell boundaries are relatively clear. | AE1/AE3 (+), Vimentin (-); Calretinin (-), MDM2 (-). |
| Malignant Mesothelioma | Epithelioid cells arranged in papillary, nested, or sheet-like patterns; cells are polygonal or cuboidal; nuclear pleomorphism is mild to moderate; cytoplasm is abundant, often with vacuoles; "window-like" spaces between cells are characteristic. | Calretinin (+), WT-1 (+), D2-40 (+), CK5/6 (+), CK7(+), AE1/AE3 (+), Vimentin (+), MDM2 (-). |
| Undifferentiated Pleomorphic Sarcoma | Markedly pleomorphic spindle and epithelioid cells; disorganized arrangement; nuclear pleomorphism is severe; mitotic figures are frequent, including abnormal mitoses; cytoplasm is scarce to moderate; no specific cellular characteristics. | Vimentin (+), AE1/AE3 (-), Calretinin (-), MDM2 (-). |

**Supplementary-Table Summary of some differential tumor cells in ascitic fluid.**

CK, cytokeratin; MDM2, mouse doubleminute 2 homolog; WT-1, Wilms tumor-1
